# Association of mutation patterns in *gyrA/B *genes and ofloxacin resistance levels in *Mycobacterium tuberculosis *isolates from East China in 2009

**DOI:** 10.1186/1471-2334-11-78

**Published:** 2011-03-29

**Authors:** Zhenling Cui, Jie Wang, Junmei Lu, Xiaochen Huang, Zhongyi Hu

**Affiliations:** 1 Shanghai Key Laboratory of Tuberculosis, Shanghai Pulmonary Hospital, Medical School, Tongji University, Shanghai 200433, China

## Abstract

**Background:**

This study aimed to analyze the association of mutation patterns in *gyrA *and *gyrB *genes and the ofloxacin resistance levels in clinical *Mycobacterium tuberculosis *isolates sampled in 2009 from East China.

**Methods:**

The quinolone resistance-determining region of *gyrA/B *were sequenced in 192 *M. tuberculosis *clinical isolates and the minimal inhibitory concentrations (MICs) of 95 ofloxacin-resistant *M. tuberculosis *isolates were determined by using microplate nitrate reductase assays.

**Results:**

Mutations in *gyrA *(codons 90, 91 and 94) and in *gyrB *(G551R, D500N, T539N, R485C/L) were observed in 89.5% (85/95) and 11.6% (11/95) of ofloxacin-resistant strains, respectively. The *gyrB *mutations G551R and G549D were observed in 4.1% (4/97) of ofloxacin-susceptible strains and no mutation was found in *gyrA *in ofloxacin-susceptible strains. The MICs of all ofloxacin-resistant strains showed no significant difference among strains with mutations at codons 90, 91 or 94 in *gyrA *(F = 1.268, *p *= 0.287). No differences were detected among strains with different amino acid mutations in the quinolone resistance-determining region of *gyrA *(F = 1.877, *p *= 0.123). The difference in MICs between ofloxacin-resistant strains with mutations in *gyrA *only and ofloxacin-resistant strains with mutations in both *gyrA *and *gyrB *genes was not statistically significant (F = 0.549, *p *= 0.461).

**Conclusions:**

Although *gyrA/B *mutations can lead to ofloxacin resistance in *M. tuberculosis*, there were no associations of different mutation patterns in *gyrA/B *and the level of ofloxacin resistance in *M. tuberculosis *isolates from East China in 2009.

## Background

Fluoroquinolones (FQs), such as ofloxacin (OFX), levofloxacin and moxifloxacin, are widely used anti-tubercular therapeutic agents for the treatment of multidrug-resistant tuberculosis (MTB) [1]. In mycobacteria, FQs bind to DNA gyrase and inhibit DNA replication [2]. This mechanism has been verified by the structural analysis and functional analysis of enzymes of *M. tuberculosis *(MTB), including DNA gyrase [3,4]. These studies showed that the MTB strains with wild-type *gyrA/B *genes were highly susceptible to FQs. Moreover, a murine model study showed that low-level FQ resistance could be overcome with the use of high dose moxifloxacin [5].

Since FQs are often prescribed as broad-spectrum antibiotics for the treatment of undiagnosed respiratory infections, and because TB patients are not treated normatively, FQ-resistant TB has become more prevalent [6]. With the occurrence of extensively drug-resistant TB in recent years, concerns about FQ-resistant TB have been raised [7-9]. Reports show that the majority (approximately 50%~90%) of FQ-resistant MTB isolates carry mutations in the quinolone resistance-determining region (QRDR) of the *gyrA *gene [2,10-15], and that a small number have mutations in the *gyrB *gene [16,17]. Much research has focused on the mutations in *gyrA/gyrB *of MTB to determine the drug susceptibility to FQs [15,18]. A correlation between quinolone susceptibility patterns and nucleotide sequences in the A and B subunits of DNA gyrase in 14 mycobacterial species has been described [19]. There are, however, no data on the correlation between quinolone susceptibility patterns and *gyrA/gyrB *mutations in MTB clinical isolates. This study aimed to investigate whether different mutations in *gyrA/gyrB *could lead to different levels of FQ-resistance in MTB strains. We analyzed the association of OFX resistance levels and the characterization of different mutations in *gyrA/gyrB *related to this resistance in clinical MTB isolates from East China.

## Methods

### Strain selection

A total of 192 MTB clinical isolates were collected from epidemiologically unlinked pulmonary TB patients in East China in 2009. The epidemiological selection was analyzed by IS6110 restriction fragment length polymorphisms [20]. All MTB strains were identified by biochemical methods and by PCR detection based on primers derived from IS6110 [21]. All samples were tested for OFX-resistance by the MGIT 960 method (Becton & Dickinson) and a total of 95 OFX-resistant MTB strains were selected at random. Ninety-seven OFX-susceptible MTB strains were also selected at random from all of the OFX-susceptible strains. The MTB H37Rv strain (ATCC 27294) was used as the reference control. The study was approved by the ethics committee of Shanghai Pulmonary Hospital. Written informed consent was obtained from all the participants.

### Drug susceptibility analysis

The drug susceptibility test (DST) of selected strains was carried out by the MGIT 960 method [22,23]. OFX (Sigma Aldrich Co., St Louis, USA) was dissolved in 0.1 M NaOH and diluted in purified water. The stock solutions were made at a concentration of 10 g/L and sterilized using a 0.22 μm polycarbonate membrane filter (Camghwohill, Co. Cork, Ireland). The stock solutions were stored at -70°C in small aliquots for up to 6 months. Frozen drug solutions were thawed once and then discarded. The OFX test concentration was 2 mg/L.

### MIC determination

The MIC determination of selected strains was carried out by using a microplate nitrate reductase assay (NRA), as described by Kumar et al [24]. The final concentrations of OFX ranged from 0.125 mg/L to 64 mg/L.

### DNA extraction

One ml of MTB suspension collected from a MGIT 960 control tube was transferred to a 1.5 ml tube and centrifuged at 10,000 g for 5 min. The supernatant was discarded and the sediment was re-suspended in 50 μl DNA extraction solution (0.04% NaOH, 0.1% SDS and 15% Chelex-100 chelating resin) and mixed by vortexing. Subsequently, the tube was incubated at 100°C for 15 min and centrifuged at 13,000 g for 10 min after it had cooled. Finally, the supernatant was transferred to another 1.5 ml tube and preserved at -20°C until further use.

### PCR

*GyrA *was amplified with the use of the *gyrA*F (5'- AGACACGACGTTGCCGC- CTG-3') and *gyrA*R (5'- CTGACCCGTTGGCCAGCAGG-3') primers. *GyrB *was amplified with the use of the *gyrB*F (5'- GCGCTGACGTCGGTGGTGAA-3') and *gyrB*R (5'- ATTCCGGGTCACTGCGCTGC-3') primers. These primers were designed by Primer-BLAST software with reference to the *gyrA *(GenBank accession no.: NC_000962.2) and gyrB (GenBank accession no.: NC_000962.2) gene sequences of MTB. The sizes of the amplified fragments were 530 bp for *gyrA *and 772 bp for *gyrB*.

### Sequencing

PCR products were purified and sequenced by Sangon Biotech (Shanghai, China). DNA sequences were analyzed with MegAlign 5.01 software (Demonstration System DNASTAR, Inc., Madison, USA).

### Statistical analysis

SPSS software (SPSS Inc., Chicago, IL, USA) was used for the statistical analysis. The MICs of OFX-resistant MTB strains with different mutations in *gyrA *or *gyrB *were compared by the analysis of variance (ANOVA) test.

## Results

Mutations were observed in the QRDRs of *gyrA/gyrB *in 87 out of 95 (91.6%) OFX-resistant MTB strains, apart from a S95T mutation in *gyrA*, which was a natural polymorphism [25] that occurred in both OFX-resistant and OFX susceptible strains in this study. No mutations were observed in the QRDRs of *gyrA/gyrB *in 8 out of 95 OFX-resistant MTB strains (8.4%) except for the S95T mutation in *gyrA*. The MICs of 97 OFX-susceptible strains were less than 2 mg/L while the MICs of 95 OFX-resistant strains were ≥2 mg/L.

Mutations were observed in the QRDR of *gyrA *in 85 of the 95 (89.5%) OFX-resistant strains, while none of the OFX-susceptible strains displayed any mutations. The most common single nucleotide mutation sites were codons 94, 91, and 90 and, in total, the relative frequencies of these codons were 56.8% (54/95), 6.3% (6/95) and 25.3% (24/95), respectively. There was one OFX-resistant strain with two mutations (codons 90 and 94) in *gyrA*.

*GyrA *mutations and MICs of the 85 OFX-resistant MTB mutants are shown in Table 1. The OFX MICs of strains with mutations at codon 94 ranged from 2 mg/L to 32 mg/L, with a median of 4 mg/L. The OFX MICs of resistant strains with mutations at either codon 90 or 91 ranged from 2 mg/L to 16 mg/L (median, 4 mg/L). The OFX MICs of three OFX-resistant groups with different single codon mutations in *gyrA *were analyzed by ANOVA, which showed no statistically significant difference among the three groups (F = 1.268, *p *= 0.287). The difference of OFX MICs of seven OFX-resistant groups with different amino acid mutations (A90V, S91P, D94A, D94G, D94N, D94Y, D94F) in *gyrA *were also analyzed by ANOVA, which also showed no significant differences (F = 0.829, *p *= 0.551). The difference of OFX MICs of five OFX-resistant groups with different amino acid mutations at codon 94 (D94A, D94G, D94N, D94Y, D94F) in *gyrA *showed no statistical significance (F = 0.455, *p *= 0.768).

**Table 1:** The patterns of *gyrA *mutations and OFX MICs profile of OFX-resistant MTB strains

Codon mutation	Nucleotide change	No. of strains				
		
		MIC (mg/L)				
		
		2	4	8	16	32
D94A	GAC-GCC	4		2		1
D94G	GAC-GGC	5	10	10	3	3
D94N	GAC-AAC	2	2	2	1	
D94Y	GAC-TAC	1	3	4		
D94F	GAC-TTC	1				
A90V	GCG-GTC/GTG	6	10	7	1	
S91P	TCG-CCG	2	2		2	
A90V&D94Y	GCG-GTC & GAC-TAC				1	

Mutations were observed in the QRDRs of gyrB in 11 of the 95 (11.6%) OFX-resistant strains. The single nucleotide mutation sites were in codons 551, 500, 539 and 485, according to the GenBank CAB02426.1 numbering system. In total, the relative frequencies of these codons were 3.2% (3/95), 4.2% (4/95), 2.1% (2/95) and 2.1% (2/95), respectively. The MICs and the mutations of *gyrA *of OFX-resistant strains with mutations in *gyrB *are shown in Table 2. There were two OFX-resistant strains with only mutations in *gyrB*, one at codon 485 and one at codon 500. Amino acid mutations were observed in QRDRs of *gyrB *in 4 of the 97 (4.1%) OFX-susceptible strains. These mutations were from Gly (GGG) to Arg (AGG) at codon 551 in three samples and from Gly (GGC) to Asp (GAC) at codon 549 in one strain. There was also a single silent mutation from Ile (ATC) to Ile (ATT) at codon 457 in one OFX-susceptible strain.

**Table 2:** The patterns of *gyrA/B *mutation and OFX MICs profile of 11 OFX-resistant MTB strains

*gyrB*		*gyrA*	No. of strains			
**Codon mutation**	**Nucleotide change**	**Codon mutation**	**MICs (mg/L)**			
			
			**2**	**4**	**8**	**16**

G551R	GGG-AGG	A90 V		1	1	
G551R	GGG-AGG	D94G		1		
D500N	GAC-AAC	A90V				1
D500N	GAC-AAC	S91P				1
D500N	GAC-AAC	D94N			1	
D500N	GAC-AAC	No mutation			1	
T539N	ACC-AAC	A90V			2	
R485L	CGT-CTT	No mutation	1			
R485C	CGT-TGT	A90V			1	

The MICs of OFX-resistant strains with mutations in *gyrA *only and without mutations in *gyrB *were compared with the MICs of OFX-resistant strains that had mutations in both *gyrA *and *gyrB*. The difference in MICs of the two groups were analyzed by ANOVA, which showed no statistical significance between the two groups (F = 0.549, *p *= 0.461).

## Discussion

Previous studies have shown that the level of drug resistance of MTB is linked to gene mutations. Huitric et al. described that most *rpoB *mutations are correlated with high-level resistance against rifampicin and that a lower level of resistance was associated with mutations in codon 522 of *rpoB *[26]. Kim et al. showed that mutations in codon 315 of *katG *were associated with high levels of isoniazid resistance, whereas a mutation in the *inhA *promoter region was associated with low-level resistance to isoniazid [27].

Mutations in short regions of *gyrA*, known as QRDR, have been associated with FQ resistance in MTB [28]. Several studies [10,11,13] have analyzed the mutations in the *gyrA *gene in clinical isolates of MTB. Most of these studies focused on the frequency of the mutations in *gyrA/gyrB *genes in FQ-resistant MTB strains. There are, however, no data on the association of mutations in *gyrA/gyrB *and FQ resistance levels in MTB isolates. Only Yin et al. have shown conclusively that different substitutions of amino acid 94 resulted in different levels of levofloxacin resistance [17].

In this study, mutations of *gyrA *were proven to be the cause of primary OFX-resistance, but an analysis of relationships between different amino acid mutations in *gyrA *and the MICs of OFX-resistant MTB strains by ANOVA showed no significant differences among the different mutants. This suggests that different amino acid substitutions at codons 90, 91 or 94 in *gyrA *bring the similar level of OFX-resistance in MTB strains, but do not contribute different level of OFX-resistance. Sun et al [29] detected the OFX MICs of laboratory-selected OFX-resistant MTB strains but did not analyze the relationship between different amino acid mutations in *gyrA *and the MICs of OFX-resistant MTB strains. We did this analysis for laboratory-selected OFX-resistant MTB strains and clinical isolates by ANOVA based on the data from their study. Our analysis showed no significant difference in the relationship between the amino acid substitution and the OFX MIC for both laboratory-selected OFX-resistant MTB strains (F = 0.091, *p *= 0.997) and clinical isolates (F = 2.023, *p *= 0.080). This is consistent with our findings in this study. However, the conclusions from both this study and the one by Sun et al. were made on only the single amino acid modification in *gyrA *in OFX-resistant isolates. The relationship between two concurrent amino acid mutations in *gyrA *and the MICs of OFX-resistant MTB strains needs to be further investigated. Our findings differed from the results of the study by Yin et al. [13], which may have been due to the different optical conformation of OFX and levofloxacin leading to different mechanisms by which OFX and levofloxacin bind to DNA gyrase A. In this study, the *gyrA *S95T was detected in all OFX-susceptible isolates and OFX-resistant isolates as a natural polymorphism, and in fact *gyrA *S95T is a phylogenetically informative polymorphism [30].

Some reports have shown that a small number of FQ-resistant MTB isolates have mutations in the *gyrB *gene, including those at codons 485, 486, 500, 509, 521, 538, 539, 540, 543 550 and 577, amongst others according to the numbering system used in this study [10,16,17,31,32]. There were only a few *gyrB *mutations that were present in FQ-susceptible MTB isolates and little evidence exists to show that mutations in *gyrB *are related to FQ resistance. In this study, a 772 bp fragment of *gyrB *was sequenced that included the codons in which mutations have been reported [10,17,31,32]. Some reported mutations, such as R485C and D500N [16,17], were also identified in OFX-resistant MTB strains in this study, but weren't detected in OFX-susceptible strains. Moreover, two OFX-resistant MTB strains in this study only carry mutations in *gyrB *at codons 485 and 500, respectively, but not in *gyrA*; the same mutations were found in MTB clinical isolates from Uzbekistan, Vietnam and Thailand [16,17,31]. In this study, the OFX-resistant MTB strain with a mutation at codon 539 in *gyrB *had a mutation A90V in *gyrA*, which was also the case in Vietnam [17]. This suggested there may be a potential relationship between these two mutations in *gyrA *and *gyrB *in the mechanism of MTB resistance to OFX. This evidence also suggests that mutations at codons 485, 500 and 539 induce resistance to OFX.

The R485L mutation found in this study differed from the R485C mutation described by Feuerriegel et al. [17], which had polymorphisms within codon 485. In this current study, the novel mutation of codon 551 was identified in both OFX-resistant and OFX-susceptible MTB strains and the novel codon 549 mutation was identified in *gyrB *in OFX-susceptible MTB strains, which supported the hypothesis that the mutations at codons 549 and 551 are not related to OFX resistance in MTB strains. Although the data from this study suggested that the mutations at codons 549 and 551 seemed not to lead to the resistance to OFX in MTB isolates, it was not possible to hypothesize whether the same mutations would have importance in the resistance of other FQs. For example, one strain that has a N533T mutation in *gyrB*, was susceptible to OFX but resistant to moxifloxacin and gatifloxacin in the study by Groll et al [32]. Although the mutation sites in *gyrB *that relate to FQ resistance are still unclear in MTB strains, the present data show that OFX-resistant strains with mutations only in *gyrA *do not differ from OFX-resistant MTB strains with mutations in both *gyrA *and *gyrB*. In this study, the relationships between mutations in *gyrB *and OFX resistance in MTB isolates remain hypothetical. We are currently performing experiments, including cloning mutations in the reference strains, which will verify the effect of *gyrB *mutations on OFX resistance in MTB strains.

## Conclusions

Our findings confirm that the susceptibility or resistance to OFX of most MTB strains can be determined by mutations in the QRDRs of *gyrA *and *gyrB*, but the level of resistance to OFX for OFX-resistant isolates could not be predicted based on the mutation patterns in the *gyrA *and *gyrB *genes. Furthermore, our findings indicate that not all the patterns of mutation in genes related to drugs reflect the resistance level of the corresponding drug for MTB drug-resistant isolates, whereas the patterns of gene mutations related to rifampicin and isoniazid resistance did reflect the resistance level of other drugs.

## Authors' contributions

Zhenling Cui designed and performed most of the experiments and dada analysis. Jie Wang performed drug susceptibility and *M. tuberculosis *identification tests. Junmei Lu carried out the MIC determination tests. Xiaochen Huang performed the PCR experiments and sequence alignment. Zhongyi Hu revised it critically and provided important scientific import. All authors approved the final manuscript.

## Competing interests

The authors declare that they have no competing interests.

## Pre-publication history

The pre-publication history for this paper can be accessed here:

http://www.biomedcentral.com/1471-2334/11/78/prepub
